# Building an Adult Congenital Heart Program: Critical Components and Important Allies

**DOI:** 10.1007/s11886-018-1080-x

**Published:** 2018-10-11

**Authors:** Akanksha Thakkar, Stephanie Fuentes-Rojas, Eunice Karanja, Ebun Ebunlomo, Allison Millette, Christine H. Lee, Y. Serena Shen-Lin, Gary Monteiro, Thomas MacGillivray, C. Huie Lin

**Affiliations:** 1 Houston Methodist DeBakey Heart & Vascular Center, 6550 Fannin Street, Suite 1901, Houston, TX 77030 USA; 2 Houston Methodist Hospital, Houston, TX USA; 3 Houston Methodist Research Institute, 6565 Fannin Street, Houston, TX 77030 USA

**Keywords:** ACHD, Business models, Transition medicine, Adult congenital cardiac surgery, Adult congenital interventional cardiology, Adult congenital cardiac anesthesia

## Abstract

**Purpose of the Review:**

The purpose of this review is to illustrate specific challenges and opportunities in the building of an adult congenital heart disease (ACHD) program and to highlight critical components and important allies.

**Recent Findings:**

With more than 1.4 million adults with congenital heart disease in the USA alone, access to specialized, compassionate, high-quality comprehensive care requires a shift toward more aggressive expansion of ACHD care, especially in the context of sparse ACHD provider representation in the vast majority of adult medical centers.

**Summary:**

The effective build of an ACHD program requires measured escalation in management of ACHD complexity matched with cultivation of key resources and clinical services ranging from congenital cardiac surgery and interventional cardiology to acquired heart disease as well as partnerships with non-cardiac specialists. By reframing ACHD care as a shared goal between patients, providers, hospitals, pharmaceutical and device industry, and payers, a potent business model can be built around the developing ACHD program to facilitate acquisition of these key resources.

## Introduction

A child born with congenital heart disease 50 years ago had a 15% chance of surviving to age 18 [1, 2], but with advances in surgical and medical therapies, there are now more adults with congenital heart disease than children [3, 4]. In the USA, there are approximately 1.4 million adult congenital heart disease (ACHD) patients with projected growth to 2.2 million by 2020 [5]; however, there are currently only 113 ACHD programs in the USA (https://www.achaheart.org/your-heart/clinic-directory/clinic-listings/). While care at ACHD centers results in better outcomes and lower mortality [6, 7], the programmatic resources required to provide excellent ACHD care are substantial (https://www.achaheart.org/media/1487/acha-achd-program-criteria-final-7116.pdf, https://www.achaheart.org/about-us/news/2017/acha-launches-national-accreditation-program/), [8, 9], as is the required institutional activation energy. The mission of providing compassionate, accessible, high-quality comprehensive adult congenital heart care is important, and the substantial effort to build and expand ACHD centers is worthwhile to fulfilling this mission.

**Fig. 1:**
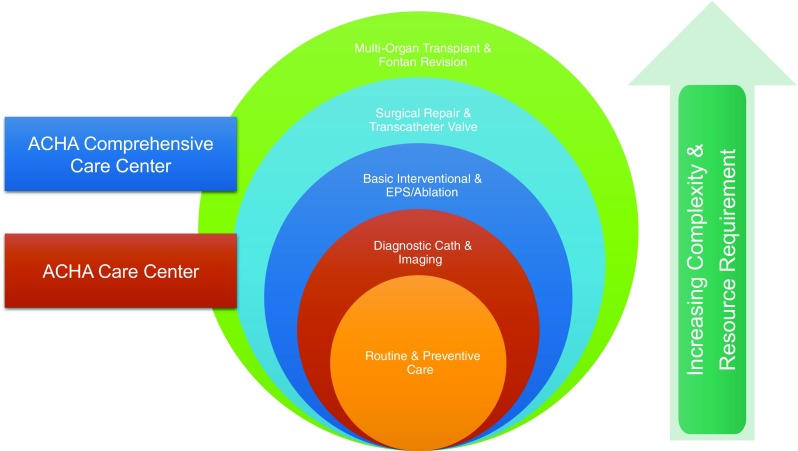
Levels of ACHD complexity care and resources requirements. Increasing complexity requires increased resources. If resources are not available for a certain level of complexity, patient safety may compel referral to a more established ACHD center

To that goal, the purpose of this manuscript is to provide suggestions to ACHD providers faced with the mammoth task of building a program. Specifically, important documents have previously described the what and why of ACHD program resources, whereas this manuscript describes how to develop and deploy ACHD resources and when they are needed for safe program growth (Fig. 1) as well as collaborations necessary for program success.

Our experience has been in building an adult congenital heart program at an adult hospital center within a seven-hospital system, and not all of our challenges will be relevant to every program. Likewise, our models may be blind to needs that other programs may have. Nevertheless, there is one absolute requirement: There must be institutional commitment to building an ACHD program, even if financial resources are limited at the start.

Finally, this manuscript aims to stimulate the savvy ACHD team’s creativity in developing and modifying business models, value propositions, and identifying synergy between customer segments. And while we emphasize the need to bring together stakeholders with shared goals and aligning incentives, the ultimate aim of these efforts is to improve care for our ACHD patients and their families.

## Building the ACHD Clinical Program

In the first month of our ACHD program, a 36-year-old man with tetralogy of Fallot who underwent repair at 1 year of life presented with a complaint of occasional “palpitations.” He had not seen a physician for 20 years. Event monitor disclosed a 38-beat run of ventricular tachycardia on day 2 of monitoring. Cardiac magnetic resonance (CMR) demonstrated severe pulmonic insufficiency and severe right ventricular (RV) enlargement.

### The Beginning: First Months of ACHD Clinic

Outpatient visits form the foundation of ACHD care as surveillance through clinical evaluation and imaging coupled with timely titration of medications is key to preventing hospital admissions and optimizing quality of life [10•]. Minimal requirements are clinic space, a clinical coordinator (nurse or medical assistant), and an ACHD cardiologist. Nevertheless, significant time investment is required to educate staff and enact patient care protocols. Pre-clinic assessment requires review of comprehensive medical records, especially operative reports which may require herculean effort to acquire, whereas clinic evaluation may require the addition of four-extremity blood pressures, pulse oximetry, posterior and right-sided EKG leads, etc., techniques that may be unusual in adult cardiology practices. Outpatient ACHD patient management requires a heart failure management infrastructure (for titrating diuretics) and management of issues ranging from arrhythmias, prosthetic valve failure, and pulmonary hypertension to infective endocarditis and pregnancy-related issues [10•]. As such, creating strong collaborations with advanced heart failure, cardiovascular imaging, electrophysiology (EP), interventional cardiology, cardiac anesthesia, and cardiovascular surgery services is also a priority. As these relationships are solidified, the ACHD cardiologist will need to recognize limitations of the program, partners, and institution and prioritize patient safety with a plan of referral to more established ACHD programs when needed (Fig. 1).

### Second Step: Committing to Invasive Interventions

Building the ACHD inpatient infrastructure is the next logical step, and committing to low complexity invasive procedures can facilitate this. These can include diagnostic catheterizations, simple transcatheter interventions [11], or EP studies/ablations and serves two major purposes: First, the program will demonstrate added value to referring physicians through ACHD-specific evaluation/treatment. Second, observation admissions for ACHD patients allow rehearsal in preparation for inpatient ACHD care and ultimately establish the inpatient infrastructure required for the care of ACHD patients. The post-cath floor may be an optimal place to start and may include basic post-procedural care, including the training of nurses, to understand acceptable oxygen saturation range, specific ACHD anatomy/physiology, alert values, and establishing lines of communication with the ACHD team. In this manner, the inpatient ACHD program can be built using elective admissions with gradual escalation of patient and procedural complexity in order to ensure institutional buy-in, resource allocation, and adequate nursing education to optimize patient safety.

### The Inadvertent Third Step: Critically Ill Admissions

In the third year of our ACHD program, a 22-year-old-man with tetralogy of Fallot was transferred with upper respiratory illness and profound cyanosis (pulse oximetry 65% on 100% non-rebreather mask). His mother related that they had left the country after his surgery at 1 month of age. Imaging demonstrated a severely stenotic 3 mm modified Blalock-Taussig-Thomas shunt and unrepaired tetralogy of Fallot. Because of undocumented immigrant status and clinical instability, the patient could not be transferred. In collaboration with a pediatric cardiac surgeon from a neighboring children’s hospital, we completed successful complete repair of tetralogy of Fallot—the first in our institution in decades.

While gradual escalation is the goal, the unpredictability of ACHD may bring critically ill patients to the program. Transfer to another more established ACHD center may be the optimal first choice, but critical illness may not permit this, and support must be requested from neighboring pediatric institutions including congenital surgical, congenital interventional, and EP physicians. These episodes of care provide case studies to educate both the hospital and outpatient practice administration to the level of resources required to care for ACHD patients and usher in the next level of complexity in the care of these patients. In this case, multidisciplinary resources were marshaled including nursing resources from the cath lab, operating room (OR), intensive care unit (ICU), and post-surgical floor, and these efforts continue to drive progression of our program.

## Nursing

### ACHD Nurse Coordinator

Nurses are the forefront of patient care coordination, including assessment, patient education, triage, diagnostic, interventional, and surgical care; the keystone provider for the ACHD program is the nurse coordinator [12]. The duties of the ACHD nurse coordinator include organizing, coordinating, and facilitating care, holistic assessment, patient education, family planning and contraception education, self-care management and self-advocacy, and advanced directive discussion. Some of these roles can be done by an exceptional medical assistant (MA) while the volume is low; however, we have found that the role quickly escalates. A survey of 15 European ACHD centers demonstrated that centers with more than 200 ACHD patients dedicated at least two ACHD nurse specialists to the practice. While there was significant variation between centers, roughly 40 patients being seen as outpatients required more than 40 h of ACHD nurse specialist time weekly, whereas each inpatient required about 3 h of nursing specialist time [13].

### Inpatient Nursing

In the last decade, simple ACHD-related inpatient admissions have doubled, and complex ACHD-related admissions have increased by 53% [14]. Admissions for non-cardiac diagnoses were associated with higher costs for moderate and severe ACHD [15]. Educating the inpatient nursing staff of a large adult hospital to provide high-quality care for this growing population of ACHD inpatients can be daunting when considering the breadth from ICUs, medical/surgical floors, emergency department, cardiac catheterization laboratory, and general and cardiac operating rooms to post-anesthesia care units. Additional training in basic physiology and anatomy of congenital cardiac lesions, the effect of cyanosis, pulmonary hypertension, and post-operative congenital heart issues is necessary [16]. Clinical nurse specialists serve as dedicated educators and can work with 2–3 nurses with special interest in ACHD to serve as champions. Mapping initial ACHD patient flow through the hospital and limiting care to certain floors can help to make the endeavor manageable. Optimizing process and nursing education during elective admissions allows for “rehearsal” between non-elective “sick patients.”

### Advanced Practice Providers

Advanced practice providers are a powerful and often underappreciated component of building an ACHD program. The nurse practitioner (NP) or physician assistant (PA) can act as a conventional physician extender by seeing ACHD patients in the inpatient and outpatient setting, providing much needed continuity. The capable NP/PA can enable the establishment of ACHD outreach clinics by providing coverage for the ACHD inpatient/consultation service or outreach services. Second, the NP/PA can optimize care coordination with inpatient and ICU nursing staff as well as outpatient services including education efforts described above. Third, they can be extremely effective in developing hospital-wide ACHD nursing protocols and competency-based education. Finally, with cultivation of leadership and management skills, experienced ACHD NP/PAs may be optimal candidates for hospital or clinical leadership positions and advocate for the needs of ACHD patients.

## Non-cardiac Consultants

ACHD providers often serve as the primary coordinator of care requiring a programmatic relationship with non-cardiac consultants including internal medicine, neurology (in particular vascular neurology), critical care, psychiatry/social work, and oral surgery/dentistry, endocrine, general surgery, and ophthalmology [17•]. In particular, obstetric and gynecologic issues in ACHD patients require meticulous multidisciplinary care coordination. In addition, the most common non-cardiac morbidities relate to the kidney (21%), lung (18%), and liver (6%) [18].

### Nephrology

Renal insufficiency is a primary driver of high resource use, responsible for 10% of ACHD hospitalizations, but up to 33% of hospital charges [19]. Mild renal insufficiency is associated with twofold increased mortality over 6 years in ACHD patients, and moderate-severe renal insufficiency is associated with a fivefold increase in mortality [20]. Renal insufficiency is common, with frank proteinuria in up to 16% of ACHD patients, which may contribute to thromboembolic risk [21]. Worse, ACHD patients may be more vulnerable to acute kidney injury through cardiac surgery and cardiopulmonary bypass [22], in particular Fontan completion [23], chronic cyanosis, and erythrocytosis [24]. Furthermore, typical algorithms to estimate glomerular filtration rate based on creatinine may underestimate renal insufficiency in Fontan patients [25]. As such, a close continuous relationship with a nephrology team is critical for optimal care of ACHD patients.

### Pulmonology

As many as 44% of ACHD patients have restrictive lung disease based on spirometry [26], and patients with single-ventricle physiology or tetralogy of Fallot may be at higher risk [27]. Scoliosis may contribute, as ACHD patients who have undergone median sternotomy in childhood may be ten times more likely to have scoliosis [28]. Reduced respiratory muscle function [29] may also contribute, as well as medication-related toxicity such as amiodarone. Indeed, patients with reduced forced vital capacity have 1.6-fold increased risk of death [30]. Similarly, obstructive sleep apnea could potentially have deleterious impact on ACHD patients [31]. Finally, complications such as hemoptysis in patients with systemic-pulmonary collaterals and plastic bronchitis in Fontan are rare but can be severely morbid. In sum, these issues underline the need for both acute and chronic support from pulmonology, especially when considering the ACHD patient with need for re-do sternotomy.

### Hepatology

ACHD patients are also at risk for acute and chronic liver complications from hepatitis, medications, or hemodynamic and physiologic perturbations. Due to absence of universal screening of donor blood, 8.6% of patients who underwent heart surgery prior to 1992 demonstrate serologic evidence of hepatitis C exposure and 4–5% may have chronic hepatitis C infection [32, 33]. Elevation of central venous pressures, hemodynamic instability, and hypoxemia contributes to the mechanisms for acute ischemic liver injury [34], explaining in part the vulnerability of this population. Similarly, over time, these insults may result in chronic injury with development of cirrhosis and subsequent evolution to hepatocellular carcinoma as seen in Fontan patients. Indeed, liver fibrosis is nearly universal both early and late after Fontan completion [35, 36]. Early screening and identification to facilitate timely treatment are important [37]. Taken together, establishing a conduit of care between the ACHD program and hepatology and even a liver transplant program is a critical component in the comprehensive care of these patients.

## ACHD as a Start-Up: Business Models, Customer Segments, and Value Propositions

Congenital heart disease is like a start-up company that has focused on innovation and development of a product for a specific customer segment (children with congenital heart disease), but success has created an even larger and fast-growing new customer segment, the ACHD patients. Like many start-up companies, ACHD programs must pivot, develop new value propositions, scale-up channels, and modify customer relationships for continued success [38] (Table 1). In addition to ACHD patients/families, the program will need to serve a number of other customer segments including hospitals, academic departments, referring physicians, and community organizations including the Adult Congenital Heart Association (ACHA) and Mended Little Hearts (MLH) [39•].

**Table 1:** Example of elements of an ACHD program business model based on Osterwalder and Pigneur [39•]

Key partners	Key activities	Value proposition	Customer relationships	Customer segments
Hospitals Academic departments Adult medicine consultants Pediatric consultants	Clinical ACHD care Transcatheter interventions EP/ablations ACHD surgery	Comprehensive ACHD care Academic productivity and fellow training Access to new patient demographic	Clinical services Telemedicine second opinion services Community	Patients/families Referring physicians Hospitals Community organizations
Cost structure	Key resources		Channels	Revenue streams
Clinical personnel Clinic infrastructure Business development Marketing	ACHD coordinator Clinical consultants Pediatric cardiology subspecialists Marketing business development		Outpatient clinics Outreach clinics Inpatient/consultation service	Clinical revenue/wRVU Downstream revenues

Both conventional and unconventional elements have been included in this non-exhaustive list

Serving so many customer segments, however, can only be beneficial if incentives are aligned, and synergy can create an environment for success [40]. For example, because the ACHD program serves as medical home to this new patient base and payer mix, access to the ACHD patients is a strong value proposition to the hospital as a significant source of downstream revenue from additional services such as imaging, oncology, orthopedics, and other traditionally high-value service lines. By providing compelling value propositions, the ACHD program can convert the hospital and academic department to key partners and justify the allocation of key resources to the program. Similarly, patients/families, employers, insurers, pharmaceutical, and device companies, as well as hospitals all ultimately benefit from timely, high-quality, specialized ACHD care, which is also part of the mission of community organizations such as the ACHA and MLH. If brought together, these customer segments can create a powerful alliance for the success of an ACHD program.

### Referring Physician Education: Culture Eats Strategy for Breakfast

Engaging and educating potential referring physicians are indispensable components to building an ACHD program. Most adult cardiology fellowships only provide minimal ACHD training [41], and teaching treatment of specific ACHD lesions is unrealistic in a single encounter, whereas, teaching indications for referral could positively impact the health of the local ACHD community [42]. Case-based teaching emphasizing specialized care of ACHD patients can impress upon referring physicians the value of the ACHD program.

In contrast, pediatric cardiologists require a different value proposition: The ACHD program can safely coordinate care in an adult hospital for ACHD patients for non-cardiac services such as orthopedics, general surgery, and oncology, as well as acquired cardiovascular disease. ACHD programs can also offer collaboration on transition as a strong value proposition.

Irrespective of the discipline, effective connection at the time of a successful meeting requires exchange of contact information (Table 2). Referring physicians must be treated with utmost respect and patience as behavior change is slow, and trust may take time to build. Ultimately, the burden is on the ACHD program to educate on added value and demonstrate that the referred patients are valued by the ACHD program.

**Table 2:** ACHD referring physician business development sample plan

	Action items	Follow-up actions
Initial meeting with potential referring physician	Education re: value proposition of ACHD program, indications for referral Exchange mobile numbers Communicate ACHD office/fax, ACHD coordinator contact (preferably via SMS contact card) Connect with referring physician’s office/business manager or referral manager	Commitment to “Just Say Yes” to all referrals and consultations Commitment to immediate accommodation of referrals
Follow-up meeting	Re-emphasize value proposition with any relevant additions Clinical updates on any shared patients (or referring physician’s partners’ patients), especially any interesting imaging or procedural findings	Identify any ACHD program issues with receiving and accommodating referrals Reinforce communication channels, especially with referring office staff
Clinical follow-up	Immediate call/text to referring physician upon evaluation of patient Immediate call/text upon completion of invasive procedure/cath/surgery and at time of hospital discharge with follow-up needs/plan	Timely transmission of consultation and/or operative report Communicate explicit description of follow-up needs and plans Create plan for “returning patient” to referring physician or co-management Optimize number of follow-up studies (e.g., TTE) that can be done in referring physician’s office

### Community Hospitals and Outreach Clinics

“Where are the ACHD patients?” is frequently asked when considering the oncoming 2.2 million US ACHD patients by 2020. Examination of US hospitals in 2017 may help: Of 5534 US hospitals, 4840 are classified as community hospitals, accounting for 780,272 beds; 33,424,253 admissions; and $902,891,035,000 of $991,531,841,000 of all hospital expenses [43]. These staggering numbers suggest that much of care for ACHD patients may be delivered in community hospitals.

System community hospitals are an excellent springboard for ACHD outreach clinics and may contribute to the build, marketing, and support of the outreach clinic as well as ACHD patient/family education and support groups. Seeing patients within their local communities contributes to patient/family satisfaction. Concerns that outreach may “dilute” ACHD care are outweighed by improvement in patient access to ACHD care. Additionally, visibility in the community markedly improves the relationship with referring physicians and may open lines of communication with emergency room, hospitalist, and intensive care physicians in the community hospital. Partnering with community cardiologists can facilitate staff education, triage, and transfer to the academic ACHD center.

With thoughtful development, outreach efforts will ultimately improve the care and outcomes of ACHD patients. As hospital system consolidation and adoption of Accountable Care Organization models accelerates, developing ACHD programs may capitalize on resources available for system integration, and the multidisciplinary nature of ACHD care will benefit from these efforts.

## Transition Care and Mental Health

“Transition” is the systematic transfer of patients from pediatric cardiology to ACHD program “to optimize the quality of life (QOL), life expectancy, and future productivity of young patients” [44]. However, only 39% of ACHD patients 18–22 years of age remain in cardiology care [45] with the first major gap in care occurring at ~ 19 years of age [46] and with men at higher risk [45, 47]. This gap can have significant consequences; 48% of late deaths in tetralogy of Fallot patients were accounted for by the 24% of who were lost to follow-up [48]. Severely complex patients were more likely to remain in care; however, financial problems and changes in insurance coverage were significant barriers, as was decreased parental involvement.

As a result, transition is a common source of anxiety among ACHD patients and their families [49]. The clinical social worker of the adult team can support transition by providing education and emotional support to the patient and caregivers, as well as by intervening in social challenges such as shifting health insurance or access to medications [50]. Return to care can be improved by physician referral [46], and transition may be facilitated by pediatric and adult practices being in the same building, shared medical records, regular outpatient reminders, efficient referrals, and nationalized health care [47]. Tools to assess transition readiness are available [51], but most importantly, transition must provide adequate time and resources.

Mental health also plays a role in the transition process, especially as mood or thought disorders are commonly present at this age. The addition of mental health disorders can intensify the process, and psychotropic medications may be necessary. Many patients and caregivers carry emotional trauma related to their medical history and require more support from the adult team both during and after transition. Counseling is beneficial to both patients and caregivers throughout ACHD care, and obtaining early evaluations by specialists and addressing mental health concerns will assist the ACHD team in supporting a patient with mental illness from transition through a healthy lifetime.

## Patient and Family Education

ACHD patients and families have important knowledge gaps [52–57], which can be exacerbated by a suboptimal transition; one study demonstrated that ACHD patients had inadequate knowledge about physical activity, reproductive health, and psychosocial matters. To fill this gap, we established the annual Houston Methodist Adult Congenital Heart Symposium patient and family education event in 2015. To further improve these education efforts, our ACHD team conducted patient focus group discussions to validate knowledge deficits, identify additional educational needs, and gather recommendations for appropriate strategies to address these needs.

During the first focus group, three major needs emerged: transition in clinical care from pediatric to adult cardiology, psychosocial aspects, and need for effective educational and outreach strategies for ACHD patients and families [49]. A second focus group described a need for more peer-to-peer communication/education, a “me too” feeling of belonging or validation, and self-advocacy.

Both focus groups highlighted the importance of social support. Participants developed a sense of camaraderie, discussed their experiences, exchanged contact information, and shared resources demonstrating the need for community level peer support, in line with efforts by the ACHA. Focus group findings have also been used iteratively to refine our annual Adult Congenital Heart Symposium, which continues to be more aligned with the needs of our patient population. Indeed, our most important finding may be the need to continuously engage local ACHD patients to understand and address specific education needs.

## Anesthesia

As the ACHD population grows, so do the number of cardiac and non-cardiac surgeries in these patients who are at increased risk for morbidity and mortality [58]; most anesthetic agents produce some vasodilation, which could cause catastrophic events in patients with intracardiac shunts, Eisenmenger, or single-ventricle physiology [59]. In one study, 55% of adverse events or death were related to intraoperative anesthetic care [58]. While repaired simple ACHD patients can be managed by most anesthesia providers, there are significant knowledge gaps when approaching complex ACHD [60].

Currently, there is no formal training program in cardiovascular anesthesia for ACHD patients. While interested fellows may spend an additional year training in congenital heart anesthesia through non-ACGME programs, adult and pediatric anesthesia residents typically have limited exposure. As such, safe perioperative management of ACHD patients can be a blind spot for growing ACHD programs. Best practice [8] and ACC/AHA guideline documents [9] recommend that anesthesia should be provided at regional ACHD centers, especially for moderate or severe congenital heart disease patients and those with advanced functional class, pulmonary hypertension, congestive heart failure, and cyanosis.

Our ACHD anesthesiologist is formally trained in both adult and pediatric cardiothoracic anesthesia with experience in mechanical support and heart/lung transplant and is supported by two other attending anesthesiologists with ACHD experience and certified nurse anesthetists with special interest in ACHD. In addition to cardiac and non-cardiac surgeries in ACHD patients, this team also participates in obstetric delivery plans on a case-by-case basis. Developing ACHD programs may consider partnering with a pediatric cardiovascular anesthesiologist. Deferring high complexity patients to other centers may be advisable during the early stages of program development.

We have found moderate-to-complex transcatheter interventions [61] to be an effective intermediate step that allowed our institution to gain experience in the perioperative management of increasing severity ACHD patients. Subsequently, we escalated to surgical and hybrid procedures in patients with significant comorbidities [62] followed by more complex surgeries and heart and heart-lung transplant in moderate complexity patients. This step-by-step escalation has enabled our ACHD anesthesia service to provide continuous excellent perioperative care.

## Cardiac Surgery: Congenital and Acquired Cardiovascular Disease

The sequelae of childhood repairs and palliations are the primary focus of ACHD surgery: intracardiac baffles, conduits, native valve repairs, and prosthetic valve replacements frequently have limited durability requiring revision or replacement in the adult. Newly diagnosed congenital lesions in adults represent a second constituency of ACHD surgery (such as sinus venosus defects with partial anomalous pulmonary venous return or anomalous origin of coronary artery).

Just as “Children are not just small adults” and “Adults are not just big children,” issues in surgical management of ACHD differ from those encountered in the pediatric as well as the acquired heart disease patient. Anatomic anomalies and surgical alterations (including shunts, baffles, and conduits) are commonly present, and review of previous operative reports is imperative in considering surgical approaches as well as multiple reentries into the chest. Primary and secondary cardiopulmonary bypass cannulation strategies should be considered in advance so that appropriate access and exposure options can be included. Native cardiac anatomy and/or surgical repair or palliation can significantly impact access to antegrade and retrograde cardioplegia, affecting myocardial protection. Frequently, reoperations can be performed on cardiopulmonary bypass with a beating heart, which often offers the best myocardial protection in complex patients. Injuries, anomalies, and acquired lesions in the coronary arteries require facility with coronary artery bypass grafting options and techniques [63]. Taken together, the surgeon, anesthesiologist, perfusionists, and intensivists require specialized training and/or experience in congenital heart disease as well as acquired heart disease surgery.

Multidisciplinary heart team decision-making including surgeons, interventionalists, advanced heart failure, imagers, and intensivists provides optimal planning and management of these complex patients. Comprehensive care should include consideration of transcatheter and hybrid techniques, EP studies/ablations, and cardiac resynchronization therapy, as well as timely escalation to durable mechanical circulatory support and heart or heart-lung transplantation. Although the periprocedural risk of transplantation in congenital heart disease patients is increased, the long-term patient and graft survivals are very favorable [64].

Finally, ACHD patients will develop acquired diseases such as diabetes, hypertension, renal failure, atherosclerosis, and chronic obstructive pulmonary disease, and as with cardiac surgery for acquired heart disease, the perioperative management of comorbidities can be challenging and impact survival. In sum, the developing ACHD program will need to gather the resources, expertise, and services to meet the complexity of each ACHD surgical patient, and judicious escalation in complexity will allow for the optimal treatment enjoyed by ACHD patients at centers of excellence specializing in ACHD [6].

## Cardiac Catheterization and Intervention

Transcatheter interventions in congenital heart disease began in 1966 with William Rashkind’s balloon atrial septostomy for transposition of the great arteries [65]. Techniques have exploded with development of various occlusion devices, stents, radiofrequency perforation wires, and transcatheter valves [66]. Anatomic and patient heterogeneity requires continuous innovation of procedures to provide patient-centered care [61, 62, 67–69], and cardiac catheterization has evolved from primarily a diagnostic modality to a vital component in the treatment of ACHD patients.

The advancement of interventional techniques has required advanced imaging to guide these procedures. A second orthogonal detector, or biplane system, allows addition of depth and is recommended for angiographic evaluation of congenital heart patients [70]. Modern cath lab systems also allow cone-beam CT through rotation of the detector system (rotational angiography) with image quality that exceeds fluoroscopy [71–73], with similar or less radiation or contrast exposure [74, 75]. Preoperative 3D datasets such as CT/MRI may also be fused with live 2D fluoroscopy and provide additional procedural guidance [69]. Both transesophageal echocardiography (TEE) and intracardiac echocardiography (ICE) are important components of ACHD transcatheter interventions [76], and fusion of echo and fluoro imaging has recently emerged to further improve interventional guidance.

Success in ACHD cath, however, relies not only on imaging and device technology, and current guidelines recommend that interventions be performed at centers with appropriate experience treating ACHD with the necessary infrastructure [9], as high volume centers have demonstrated better outcomes [77]. Developing the needed infrastructure requires meticulous staff training, outfitting equipment, and adopting standard congenital heart catheterization protocols; we found that adoption of a dedicated ACHD cath team is required to surmount the necessary learning curve and may reduce case time by up to one third (Fuentes et al. submitted manuscript). Finally, the developing ACHD program will need to recruit or collaborate with experienced congenital operators if not available within the institution.

## Conclusion

The need for specialized ACHD care is a rapidly growing public health problem, and the majority of adult health care facilities in the USA may not be able to provide for this need. The continued development of safe and accessible ACHD care must be a priority for the ACHD provider community, and incumbent to this process is the ability to create safe, sustainable growth while acquiring needed resources coordinated with escalation in ACHD patient complexity. Continued study of these resource needs and ACHD program building is an important priority to achieve these goals as is the need to train ACHD providers accordingly.

## Abbreviations

ACHD: Adult congenital heart disease
EP: Electrophysiology
EKG: Electrocardiogram
RV: Right ventricle
CMR: Cardiovascular magnetic resonance
OR: Operating room
ICU: Intensive care unit
MA: Medical assistant
NP: Nurse practitioner
PA: Physician’s assistant
ACC: American College of Cardiology
AHA: American Heart Association
CT: Computed tomography
TEE: Transesophageal echo
ICE: Intracardiac echo
